# German Hospital, Dalston

**Published:** 1910-12-31

**Authors:** 


### GERMAN HOSPITAL, DALSTON.

### NEW BLOCK FOR NURSING SISTERS AND SERVANTS.

This block is to give accommodation for twenty-one nursing sisters and sixteen female servants on the ground floor and on the first and second floors, while in the basement it provides room for the engineer, for the boiler house, and for hospital storerooms. It is proposed to erect the block on the southern boundary of the hospital grounds, and the site now occupied by houses known as No. 5 and 6 Clifton Grove, N.E., which are the property of the hospital, and which are to be demolished. A side passage, 5 feet wide, between No. 4 Clifton Grove and the new block, will be provided for the purpose of receiving fuel and hospital stores.

The basement of the new building is to be 5 ft. 3 in. below the ground level, and the entrance 5 ft. 3 in. above the ground level, giving a clear height in the basement of 9 ft. 6 in., in the ground floor of 11 ft., in the first floor of 10 ft. 6 in., and in the second floor of 10 ft. 3 in.

The ground floor is planned to comprise a large sitting-room for the use of the nursing staff, and ten single bedrooms, with bath, lavatories, w.c., housemaids' sink, and cupboards.

On the first floor there will be a sitting-room for female servants, and eight single bedrooms for nurses, with a larger room for three night nurses, with bath, etc., as on ground floor, while the second floor will comprise sleeping accommodation for sixteen female servants in six rooms, also bathroom, lavatories, w.c., housemaids' sink, and cupboards.

The basement floor will be in concrete with granolithic cement paving, while all the other floors and staircase will be in steel and concrete construction, the corridors with 2 in. "coved" corners. The sitting-rooms and bedrooms are to have 1½ in. hard maple wood-block flooring with coves against the walls. The plastering will be finished in Keene's cement with 2 in. coves against ceilings, and with rounded window-sills. The staircase will be lined with teak, and will have balusters, newels, and handrail of the same material. The glazed screen with swing doors on first and second floor landings will be in teak, and the sash doors and fanlights at end of corridors near fire-escape staircase will be of the same wood.

The Mansard roof will be constructed of wood, covered with boards and felt outside, and with slate to match existing buildings. The walls are to be built with red-facing bricks and white Gault bricks, as used in existing buildings. The entrance, portico stone steps, and window-sills to be in Portland stone or York stone. Bathrooms, lavatories, w.c.'s, and housemaids' sinks are to be lined with white glazed tiles; the corridors and landings and staircase to have painted dados 4 ft. 6 in. high.

The sitting-rooms will have open fireplaces, while all the rooms and corridors and landings will be heated by means of hot-water radiators. The building will be lit by electricity.

The building contract has been undertaken by Messrs. Foster and Dicksee, of Rugby, who have already commenced the work.

